# The Impact of Environmental Benzene, Toluene, Ethylbenzene, and Xylene Exposure on Blood-Based DNA Methylation Profiles in Pregnant African American Women from Detroit

**DOI:** 10.3390/ijerph21030256

**Published:** 2024-02-23

**Authors:** Jennifer K. Straughen, Ian Loveless, Yalei Chen, Charlotte Burmeister, Lois Lamerato, Lawrence D. Lemke, Brendan F. O’Leary, John J. Reiners, F. Gianluca Sperone, Albert M. Levin, Andrea E. Cassidy-Bushrow

**Affiliations:** 1 Department of Public Health Sciences, Henry Ford Health, 1 Ford Place, Detroit, MI 48202, USAllamera1@hfhs.org (L.L.); alevin1@hfhs.org (A.M.L.); acassid1@hfhs.org (A.E.C.-B.); 2 Department of Obstetrics, Gynecology and Reproductive Biology, College of Human Medicine, Michigan State University, East Lansing, MI 48824, USA; 3 Henry Ford Health + Michigan State University Health Sciences, Detroit, MI 48202, USA; 4 Department of Earth and Atmospheric Sciences, Central Michigan University, Brooks Hall 314, Mount Pleasant, MI 48859, USA; l.d.lemke@cmich.edu; 5 Department of Civil and Environmental Engineering, Wayne State University, 2100 Engineering Building, Detroit, MI 48202, USA; ax9873@wayne.edu (B.F.O.); fgsperone@wayne.edu (F.G.S.); 6 Department of Biology, Wayne State University, 5047 Gullen Mall, Detroit, MI 48202, USA; 7 Center for Urban Responses to Environmental Stressors, Wayne State University, 6135 Woodward Ave, Detroit, MI 48202, USA; ad5180@wayne.edu; 8 Institute of Environmental Health Sciences, Wayne State University, 6135 Woodward Ave, Detroit, MI 48202, USA; 9 Department of Environmental Science and Geology, Wayne State University, 4841 Cass Avenue, Detroit, MI 48201, USA; 10 Department of Pediatrics and Human Development, College of Human Medicine, Michigan State University, East Lansing, MI 48824, USA

**Keywords:** DNA methylation, pregnancy, BTEX, epidemiology, volatile organic compounds

## Abstract

African American women in the United States have a high risk of adverse pregnancy outcomes. DNA methylation is a potential mechanism by which exposure to BTEX (benzene, toluene, ethylbenzene, and xylenes) may cause adverse pregnancy outcomes. Data are from the Maternal Stress Study, which recruited African American women in the second trimester of pregnancy from February 2009 to June 2010. DNA methylation was measured in archived DNA from venous blood collected in the second trimester. Trimester-specific exposure to airshed BTEX was estimated using maternal self-reported addresses and geospatial models of ambient air pollution developed as part of the Geospatial Determinants of Health Outcomes Consortium. Among the 64 women with exposure and outcome data available, 46 differentially methylated regions (DMRs) were associated with BTEX exposure (FDR adjusted *p*-value < 0.05) using a DMR-based epigenome-wide association study approach. Overall, 89% of DMRs consistently exhibited hypomethylation with increasing BTEX exposure. Biological pathway analysis identified 11 enriched pathways, with the top 3 involving gamma-aminobutyric acid receptor signaling, oxytocin in brain signaling, and the gustation pathway. These findings highlight the potential impact of BTEX on DNA methylation in pregnant women.

## 1. Introduction

Increasingly, studies are linking air pollution to an increased risk of adverse pregnancy outcomes such as preterm birth (PTB) [1]. Among these pollutants, volatile organic compounds such as BTEX (benzene, toluene, ethylbenzene, and xylenes) are of concern as they are widespread in urban areas [2]. BTEX, commonly released from petroleum products and vehicle exhaust, has been found in soil and groundwater at both commercial and residential properties [3,4,5]. We have previously shown that BTEX in the airshed is associated with an increased risk of PTB in Detroit [1]. Given the widespread distribution of BTEX and its potential effects on pregnancy outcomes, it is important to explore potential biomarkers and pathways by which BTEX might influence maternal prenatal health and pregnancy outcomes.

Epigenetic changes, namely DNA methylation, has emerged as a potential mechanism by which volatile organic compound exposure may exert adverse health effects [6,7,8]. Most of these studies have focused on exposure to a single component of BTEX (e.g., benzene [9]) or occupationally exposed individuals, and none have studied the association of these compounds with DNA methylation in pregnancy [10]. For example, exposure to BTEX as a consequence of being a gas station worker was associated with hypermethylation in the promoter region of two tumor suppressor genes and one detoxification gene [11]. Additionally, one study found that even lower levels of benzene exposure (<1 part per million) was associated with alterations in genes involved in the innate immune response and energy homeostasis [12]. To our knowledge, the impact of BTEX exposure on genome-wide methylation changes in DNA from maternal blood has not been examined. Given this evidence demonstrating that even lower exposure levels may impact epigenetic changes, it is important to examine BTEX exposure and DNA methylation among more generally exposed groups of individuals, including non-occupationally exposed pregnant women.

This study sought to examine the association of airshed BTEX with genome-wide methylation changes in DNA from maternal prenatal blood specimens from a cohort of African American women residing in an urban area. In the United States as a whole and in Detroit, MI, African American women bear a disproportionate burden of both environmental toxicant exposures and adverse health outcomes such as PTB [13]. As such, there is an immediate need to include minorized groups in research. By focusing on African American women in this analysis, we may reveal potentially meaningful biomarkers or pathways or areas for intervention to decrease racial disparities in key health outcomes.

## 2. Materials and Methods

### 2.1. Study Population

This study used questionnaires, medical record data, and venous-blood-derived DNA from the Maternal Stress Study (MSS), which recruited African American women, aged 18–44 years, in the second trimester of pregnancy between February 2009 and June 2010 [14]. All women were seeking prenatal care at Henry Ford Health System in Detroit, Michigan. In total, 203 women participated in the MSS. A subset of women in the MSS resided in the study area that was included in the Geospatial Determinants of Health Outcomes Consortium (GeoDHOC) study and had BTEX exposure data available from the GeoDHOC (n = 142) study as described below. After further restricting to women who had archived DNA that was previously extracted from a second-trimester blood specimen, the final analytic sample included 64 women who had both exposure and outcome data available. This study was approved by the institutional review board at Henry Ford Health System #5316 from 28 October 2008 to present and #7777 from 9 August 2013 to present. Written informed consent was obtained for all study participants as part of #5316.

### 2.2. BTEX Exposure Assignment

BTEX exposure was estimated through high-resolution spatial monitoring of air pollution by the GeoDHOC, which has been described in detail elsewhere [1,15,16]. The GeoDHOC study collected air pollution measurements at 100 sites in 2008 and 133 sites in 2009 in Detroit and Windsor during 2 separate 2-week campaigns (September 2008 and May–June 2009) [1,15,16]. GeoDHOC geospatial models of Detroit air pollution [15] were coupled with pollution data collected by the State of Michigan Air Sampling Network in Detroit to develop a model that estimates ambient Detroit air pollution at a spatial density of 300 square meters [17]. Kriging was used to model individual air pollutant content across Detroit based on maternal address. The MSS recruitment partially overlapped with the GeoDHOC sampling frame, facilitating exposure estimation in a subset of the MSS cohort. Trimester-specific exposure to airshed BTEX was estimated using the GeoDHOC data described previously [14]. In brief, maternal address at the time of a second trimester blood draw was self-reported and was the address used to assign BTEX exposure for that trimester. The date of a participant’s last menstrual period was used to define each trimester of pregnancy which was then used to assign exposure periods. For this study, we focused on exposures during the second trimester of pregnancy. Exposure estimates for pregnancies with a last menstrual period date on or before the 15th of each month were considered to begin in that month while exposures for pregnancies with a last menstrual period date after the 15th of a month were considered to begin the following month [1]. The average exposure estimates during the months corresponding to the second trimester were then calculated. For the primary analysis, BTEX was dichotomized into low versus high second trimester BTEX exposure using the median BTEX level in the sample (median = 7.42 μg/m^3^; range = 6.02–11.32 μg/m^3^).

### 2.3. DNA Isolation and Measurement of DNA Methylation

Maternal DNA was isolated from a venous blood draw during the second trimester of pregnancy and has been stored at −80 °C since the time of extraction. Bisulfite conversion was performed with the EZ-96 Methylation Kit (Zymo Research, Irvine, CA, USA) using the standard methods. The Illumina Infinium MethylationEPIC BeadChip (EPIC; San Diego, CA, USA) was then used to measure genome-wide DNA methylation at the Wayne State University Genome Sciences Core Laboratory.

### 2.4. Methylome-Wide Assessment and Quality Control 

Illumina EPIC Array DNA methylation data were generated for 64 unique pregnant mothers with archived DNA. The R package “SeSAMe” was used to initially process the over-850,000 CpG probes on the array by performing background adjustment, dye bias correction, and masking of low-intensity probes, as well as probes with design issues (e.g., overlap with single-nucleotide polymorphisms) [18]. CpG sites with all patient probes masked were excluded, leaving 749,000 probes for the maternal blood data analysis. A Beta-Mixture Quantile approach was used to normalize the type II CpG probes to the type I CpG probes, removing bias associated with technical variation [19]. Remaining CpG site missing values were imputed using a sliding logistic regression in which probes with complete data within 1 Mb in either direction of a given CpG site were used for model construction [20]. A logit transformation was used to convert methylation Beta values to M-values, which were used for association analysis.

Additionally, maternal blood methylation data were deconvoluted to their estimated constituent cell types using the Houseman method [21] as part of the “minfi” package [22]. Specifically, immune-cell-specific proportions were estimated for each participant, and these estimates were utilized as covariates in the methylation analyses to account for potential confounding by differential distributions of cell types.

### 2.5. Covariate Assessment

MSS participants self-reported date of birth (used to calculate maternal age at time of blood draw), marital status, education, cigarette smoking (self and environmental tobacco exposure), and pre-pregnancy height and weight. Pre-pregnancy body mass index (BMI) was calculated as weight (kg)/height (m^2^). Gestational age at delivery, parity, offspring birth weight, and sex were abstracted from the medical record.

### 2.6. Statistical Analysis

The primary analysis for this investigation was a differentially methylated region (DMR)-based epigenome-wide association study of second-trimester BTEX exposure. This analysis involved 2 stages, with the first stage composed of single-CpG-site association testing and the second stage combining the single-site association results to perform the region-based analysis. Specifically, the first stage was performed using linear regression to test the association between each CpG site M-value and second-trimester BTEX (high vs. low), while adjusting for the following potential confounding variables: maternal age, maternal pre-pregnancy BMI, and methylation-based estimates of blood cellular composition proportions (described above). A *p*-value threshold of <0.01 was used as the cutoff to advance individual CpG sites to the region testing in stage 2. The stage 2 identification of DMRs associated with BTEX exposure was performed using the comb-p method as implemented in the R package “ENmix” [23,24]. Specifically, comb-p computes a region-based test of association by combining the single CpG site association *p*-values from stage 1 where the CpG sites reside within a 1 kb range of one another. An epigenome-wide permutation-based approach was then applied to determine the *p*-value for each region. For this analysis, the resulting region-based *p*-values were then adjusted for multiple testing using the Benjamini and Hochberg false discovery rate (FDR) approach [25], and DMRs with an FDR adjusted *p*-value < 0.05 were considered statistically significant. Significant regions were annotated to genes using the ChIPseeker R package with default parameters and the UCSC hg38 knownGene Track as reference [26]. All CPG sites that were statistically significant (FDR adjusted *p*-value < 0.05) and an absolute log-fold change >1 were subsequently used to test for biological pathway enrichment using QIAGEN Ingenuity Pathway Analysis (Hilden, Germany) [27].

## 3. Results

Sociodemographic and reproductive characteristics of the 64 women overall and by low/high BTEX exposure are presented in Table 1. The mothers had an average age of approximately 26 years, the majority (56%) had a prior pregnancy resulting in a live birth, 36% had more than a high school education, and 19% were married. Higher BTEX levels were associated with a lower pre-pregnancy BMI (*p* = 0.047) and younger maternal age (*p* = 0.009).

**Figure 1:**
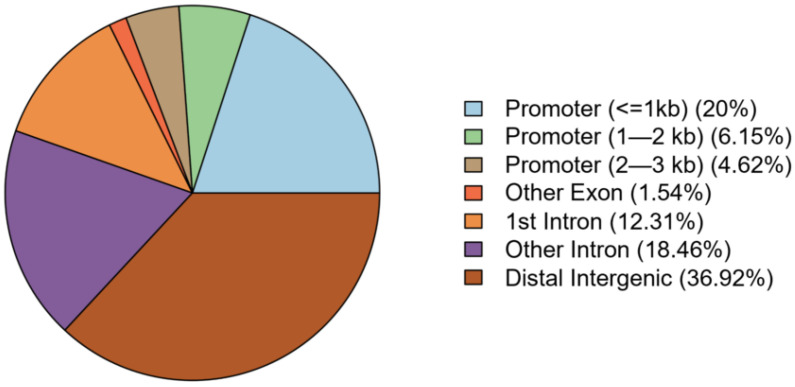
Distribution of the functional annotation categories of the DMRs associated with BTEX exposure.

A total of 46 DMRs (FDR adjusted *p* < 0.05) were associated with BTEX exposure (Table 2), and these DMRs included a total of 201 independent CpG sites. On average, DMRs spanned 236 base pairs (minimum/maximum base pair width 4/911), and while they were spread throughout the genome, chromosome 11 contained the highest number of DMRs (n = 6). The distribution of the functional annotation categories of the DMRs are presented in Figure 1, and these data show that 63.08% of the DMRs were in genic regions (exon, introns, or promoters) of the genome, while the remaining 36.92% were considered distal intergenic. Further, based on the nominally significant (single CpG site *p*-value < 0.05) CpGs within each of the DMRs, there was a general trend towards consistent association directions with increasing BTEX exposure for the DMRs. Specifically, 41 of the DMRs (89%) had nominally significant CpGs where a higher BTEX exposure was associated with hypomethylation, while a higher BTEX was associated with hypermethylation only at a single region on chromosome 10 (annotated as distal intergenic, with the nearest gene being a long non-coding RNA, *LINC02681*). The remaining four regions (9% of DMRs) were composed of a similar number of both BTEX-associated hyper- and hypo-methylated CpGs.

In Table 2, the top 5 most significant regions mapped to genes on chromosome 4 (long intergenic non-protein coding RNA 1093, FDR = 1.67 × 10^−9^), chromosome 6 (dual specificity phosphatase 22, FDR = 1.76 × 10^−9^), chromosome 11 (cholecystokinin B receptor, FDR = 1.76 × 10^−9^), chromosome 17 (WAS/WASL interacting protein family member 2, FDR = 1.02 × 10^−7^), and chromosome 1 (ribosomal modification protein rimK like family member A, FDR = 2.23 × 10^−7^). Higher BTEX levels were associated with hypomethylation within all of these regions. Also, biological pathway analysis was performed on the set of genes annotated to each of the significant DMRs using Ingenuity Pathway Analysis. Ingenuity Pathway Analysis identified 11 canonical pathways that were nominally (*p* < 0.05) enriched for these genes. These pathways are included in Table 3. The top 3 canonical pathways included functions in gamma-aminobutyric acid (GABA) receptor signaling (*p* = 0.002), oxytocin in brain signaling pathway (*p* = 0.006), and the gustation pathway (*p* = 0.007).

## 4. Discussion

This study found that African American women residing in an urban area with higher second-trimester airshed BTEX exposure had altered DNA methylation in 46 DMRs in blood from the second trimester. In addition, results suggest that increasing BTEX exposure tended to be associated with hypomethylation in these DMRs. Several biologic pathways with plausible relevance to pregnant women and the risk of adverse pregnancy outcomes were identified.

Our study adds to the growing body of research demonstrating that exposure to volatile organic compounds, such as BTEX, alters DNA methylation [11,28]. Exposure to BTEX, assigned based on occupation as a gas station worker (exposed), compared to other work was associated with hypermethylation in the promoter region of p14ARF and p16INK4A [11]. p14ARF and p16INK4A are both located in the *CDKN2A* gene and are involved in cellular senescence and aging [29]. Our study did not a priori examine methylation at these sites and focused on pregnant women where exposure was defined using their residential address. Compared to occupational exposure, airshed BTEX exposure is likely lower, which may explain why these sites were not identified as statistically significant DMRs in the present analysis.

Other studies that examined components of BTEX, but not the entire mixture, also reported associations with DNA methylation. Toluene, ethylbenzene, and xylene exposure was associated with significant downregulation of the expression of genes involved in cell-mediated immune and inflammatory responses [30]. In this same study, exposure-related changes in gene expression were also associated with changes in DNA methylation [30]. Schiffman et al. found that lower benzene exposure (<1 part per million) was associated with alterations in genes involved in the innate immune response and energy homeostasis [12]. Similarly, trichloroethylene exposure is associated with methylation changes in genes related to cell–matrix adhesion and interferon expression [31]. Importantly, the aforementioned studies were in non-pregnant populations and we focus here on pregnant African American women.

Our study is unique in that it examined changes in DNA methylation in a cohort of pregnant women; thus, the changes observed here may be important for pregnancy maintenance and parturition. We previously showed that higher ambient BTEX exposure in pregnancy is associated with an elevated risk of PTB in Detroit [1]. That study together with the results presented here suggest pathways and potential biomarkers to explore in future studies. Of note, 2 of the top 3 canonical pathways identified as significant in this analysis are associated with labor and preterm labor. GABA receptor signaling and oxytocin in brain signaling pathways were both previously identified to be associated with labor and pregnancy, and dysregulation or changes in these pathways may contribute to the early onset of labor or parturition [32,33,34]. Pregnancy maintenance is a well-orchestrated series of integrated events, and disruption of this fine-tuned process can result in spontaneous abortion, PTB, or other adverse outcomes. As such, the findings here might represent pathways by which BTEX and related exposures might contribute to suboptimal pregnancy outcomes like PTB, which is significantly higher among African American women. Studies with a larger sample size are needed to test whether the associations between BTEX and PTB are mediated by DNA methylation changes. Interestingly, previous studies have found an association between volatile organic compounds and some of the same pathways that were significant in this study. For example, toluene, a BTEX component, is associated with GABA signaling in other systems (brain) [35]. In addition, exposure to polycyclic aromatic hydrocarbon (a semi-volatile organic compound) was found to be associated with the hypomethylation of *DUSP22* [36]. Together, these previous studies support our current findings.

This study has several strengths including detailed estimates of maternal exposure to ambient BTEX. In addition, the inclusion of African American women in this study is particularly poignant as African American women in the United States have some of the highest rates of adverse pregnancy outcomes [13,37]. Despite this, several limitations merit mention. First, this study had a relatively small sample size. In addition, our analyses only considered maternal residential addresses reported at the time of biospecimen collection (second trimester). We could not consider the length of time spent at other addresses (e.g., places of employment) or relocation during pregnancy, both of which could have resulted in the misclassification of BTEX exposure. Similarly, we did not have information on indoor BTEX exposures, which can be higher than outdoor ambient levels. As such, the true BTEX exposure level might be different from the estimated exposure used in this study. Additional studies that consider total BTEX exposure (exposures from all sources), such as through measurement of BTEX metabolites in urine, are needed to further understand the health effects of BTEX exposure, including non-occupational exposures. We recognize that DNA methylation in the blood may not be the target tissue for BTEX exposure; thus, future studies could consider studying DNA methylation in placental tissue which may have a direct impact on pregnancy outcomes. It is also possible that these findings were impacted by cell type distribution as peripheral blood DNA represents a composite of cell types, but we used a deconvolution method to account for potential bias [21].

## 5. Conclusions

This analysis demonstrates that BTEX exposure is associated with changes in DNA methylation and that there is a general trend of higher BTEX exposure being associated with hypomethylation. From a functional perspective, several of the identified sites are important for pregnancy and labor. Considered together, these findings suggest a mechanism by which BTEX exposure may increase the risk of PTB and may present an important avenue for future research and prevention of PTB.

## Figures and Tables

**Table 1:** Maternal and infant demographic and clinical characteristics overall and stratified by median BTEX exposure (median = 7.42 μg/m^3^).

	Combined Population (N = 64)	Low BTEX (N = 32)	High BTEX (N = 32)	*p*-Value
Maternal education (≥ college), n (%)	20 (36.4)	7 (28.0)	13 (43.3)	0.370
Maternal age at blood draw, mean (SD)	25.84 (5.89)	27.72 (6.49)	23.95 (4.59)	0.009
Pre-pregnancy BMI (kg/m^2^), mean (SD)	28.13 (6.66)	29.77 (6.24)	26.48 (6.76)	0.047
Married, n (%)	12 (18.8)	8 (25.0)	4(12.5)	0.337
Prenatal smoking, n (%)	7 (10.9)	3 (9.4)	4 (12.5)	1.000
ETS exposed, n (%)	21 (70.0)	13(76.5)	8 (61.5)	0.630
Parity, mean (SD)	1.05 (1.43)	1.09 (1.12)	1.00 (1.70)	0.796
Methylation-based predicted immune cell %, mean (SD)				
CD8+ T-cell	8.97 (3.77)	8.39 (2.90)	9.55 (4.44)	0.219
CD4+ T-cell	7.11 (3.38)	7.68 (3.35)	6.54 (3.36)	0.181
NK-cell	1.69 (2.37)	1.57 (1.46)	1.82 (3.05)	0.681
B-cell	3.10 (1.69)	3.19 (1.84)	3.02 (1.54)	0.696
Monocytes	9.64 (2.27)	9.59 (2.12)	9.69 (2.45)	0.870
Granulocytes	69.48 (7.00)	69.59 (6.13)	69.38 (7.87)	0.909
Infant sex (male), n (%)	33 (52.4)	18 (56.2)	15 (48.4)	0.710
Birthweight (grams), mean (SD)	3151.29 (610.96)	3182.22 (605.63)	3119.35 (624.76)	0.687
Gestational age at delivery (weeks), mean (SD)	38.73 (2.47)	38.81 (1.99)	38.64 (2.90)	0.780

BMI, body mass index; BTEX, benzene, toluene, ethylbenzene, and xylenes; ETS, environmental tobacco smoke; SD, standard deviation. *p*-value from *t*-test for continuous variables and from chi-square test for categorical variables.

**Table 2:** Significant differentially methylated regions (DMRs) in second-trimester maternal blood specimens comparing high vs. low BTEX exposure. DMRs are arranged by increasing FDR *p*-value.

						CpG Sites *		
Chromosome	BP Start	BP Width	Functional Annotation	Gene Symbol	FDR	Total	Hyper	Hypo
4	184,908,253	766	Distal intergenic	*LINC01093*	1.67 × 10^−9^	11	0	10
6	291,686	911	Promoter (≤1 kb)	*DUSP22*	1.76 × 10^−9^	8	0	8
11	6,291,624	888	Distal intergenic	*CCKBR*	1.76 × 10^−9^	7	0	7
17	40,274,523	289	Promoter (≤1 kb)	*WIPF2*	1.02 × 10^−7^	7	0	6
1	42,384,283	365	Intron	*RIMKLA*	2.23 × 10^−7^	8	0	8
7	69,064,092	87	Distal intergenic	*CT66*	3.26 × 10^−7^	4	0	4
2	48,132,739	315	Intron	*FOXN2*	3.26 × 10^−7^	7	0	7
17	80,408,535	394	Intron	*RNF213-AS1*	3.30 × 10^−7^	8	0	6
21	47,532,059	275	Distal intergenic	*PRMT2*	6.89 × 10^−7^	5	0	4
12	123,319,893	165	Exon	*SBNO1*	8.02 × 10^−7^	5	0	4
14	93,698,773	172	Intron	*UNC79*	8.02 × 10^−7^	3	0	3
11	123,430,574	376	Promoter (≤1 kb)	*GRAMD1B*	8.67 × 10^−7^	6	0	6
1	161,008,461	366	Intron	*F11R*	8.90 × 10^−7^	8	3	3
12	123,750,781	84	Promoter (≤1 kb)	*ATP6V0A2*	9.13 × 10^−7^	3	0	3
5	170,814,528	309	Distal intergenic	*GABRP*	9.13 × 10^−7^	8	0	8
19	2,163,592	241	Promoter (≤1 kb)	*DOT1L*	1.01 × 10^−6^	4	0	4
11	9,697,192	238	Intron	*SWAP70*	1.03 × 10^−6^	2	1	1
17	53,828,262	254	Intron	*KIF2B*	1.03 × 10^−6^	6	0	4
19	35,645,555	158	Promoter (1–2 kb)	*ETV2*	1.03 × 10^−6^	8	0	6
16	56,228,384	361	Promoter (2–3 kb)	*GNAO1*	1.87 × 10^−6^	9	0	7
3	9,932,179	145	Promoter (1–2 kb)	*IL17RC*	1.93 × 10^−6^	6	0	4
2	48,844,762	307	Intron	*STON1-GTF2A1L*	2.31 × 10^−6^	6	0	5
22	44,422,011	189	Distal intergenic	*LINC01656*	2.46 × 10^−6^	5	3	2
16	10,837,596	110	Distal intergenic	*TVP23A*	2.71 × 10^−6^	8	0	6
6	85,823,948	269	Distal intergenic	*SNHG5*	4.21 × 10^−6^	4	0	4
11	117,352,729	211	Promoter (≤1 kb)	*CEP164*	5.14 × 10^−6^	4	0	4
14	105,287,325	103	Intron	*BRF1*	5.46 × 10^−6^	3	0	3
19	37,825,319	254	Promoter (2–3 kb)	*LOC644554*	6.11 × 10^−6^	6	0	6
10	133,938,603	291	Distal intergenic	*FRG2B*	6.18 × 10^−6^	5	0	3
3	15,469,026	292	Intron	*COLQ*	8.95 × 10^−6^	6	0	4
19	49,222,966	288	Distal intergenic	*TRPM4*	1.16 × 10^−5^	4	0	3
11	116,658,839	232	Distal intergenic	*LINC02702*	3.16 × 10^−5^	5	0	5
7	95,064,396	79	Intron	*PPP1R9A*	3.97 × 10^−5^	5	0	4
16	1,199,498	27	Intron	*CACNA1H*	4.70 × 10^−5^	2	0	2
9	130,533,824	27	Distal intergenic	*FUBP3*	6.41 × 10^−5^	4	0	2
16	1,031,442	17	Distal intergenic	*SSTR5-AS1*	7.07 × 10^−5^	2	0	2
16	66,638,395	205	Promoter (1–2 kb)	*CMTM4*	1.27 × 10^−4^	6	0	4
16	12,070,415	274	Intron	*SNX29*	1.42 × 10^−4^	5	0	4
19	44,259,187	10	Promoter (≤1 kb)	*ZNF233*	1.44 × 10^−4^	2	0	2
10	101,282,815	69	Distal intergenic	*LINC02681*	2.29 × 10^−4^	2	2	0
12	111,126,997	143	Intron	*CUX2*	6.16 × 10^−4^	4	1	1
1	166,958,580	4	Intron	*ILDR2*	0.001	2	0	2
3	88,198,600	152	Distal intergenic	*C3orf38*	0.002	4	0	1
11	368,564	75	Promoter (≤1 kb)	*B4GALNT4*	0.003	5	0	2
2	8,597,158	31	Distal intergenic	*LINC01814*	0.003	3	0	2
2	1846836	18	Intron	*MYT1L*	0.004	2	0	2

BP, base pair; BTEX, benzene, toluene, ethylbenzene, and xylenes; FDR, false discovery rate. BP Start denotes the 5’ most base pair position within each region based on genome build hg38. BP Width is the number of base pairs over which each region spans. Functional Annotation corresponds to the annotation category assigned to each region by CHiPseeker. Gene Symbol is the official gene symbol for the gene nearest to the region identified by CHiPseeker. False discovery rate is the adjusted *p*-value for the region as determined by comb-p. * The total number of CpG sites within each DMR, and the subset with single CpG site nominal *p*-values < 0.05, where increasing BTEX exposure was associated with hyper- and hypo-methylation.

**Table 3:** Significant canonical pathways from Ingenuity Pathway Analysis based on significant genes from region-based analysis, associated with high vs. low BTEX exposure.

Ingenuity Canonical Pathways	*p*-Value	Proportion	Genes
GABA Receptor Signaling	0.00219	0.023	*CACNA1H, GABRP, GNAO1*
Oxytocin In Brain Signaling Pathway	0.00646	0.015	*CACNA1H, GNAO1, NLRP5*
Gustation Pathway	0.00708	0.015	*CACNA1H, GABRP, TRPM4*
Assembly of RNA Polymerase III Complex	0.02510	0.077	*BRF1*
Role of Macrophages, Fibroblasts and Endothelial Cells in Rheumatoid Arthritis	0.02570	0.009	*GNAO1, IL17RC, RIPK1*
G Beta Gamma Signaling	0.02570	0.016	*CACNA1H, GNAO1*
Role of IL-17A in Psoriasis	0.02690	0.071	*IL17RC*
Endocannabinoid Neuronal Synapse Pathway	0.03390	0.013	*CACNA1H, GNAO1*
Corticotropin Releasing Hormone Signaling	0.03470	0.013	*CACNA1H, GNAO1*
Androgen Signaling	0.04270	0.012	*CACNA1H, GNAO1*
IL-17A Signaling in Gastric Cells	0.04900	0.039	*IL17RC*

BTEX, benzene, toluene, ethylbenzene, and xylenes; GABA, gamma-aminobutyric acid; IL, interleukin. The proportion column is the proportion of genes that were identified as significant that were present in the pathway.

## Data Availability

The data presented in this study are available on request from the corresponding author.
